# Video-assisted thoracoscopic surgery in the management of penetrating and blunt thoracic trauma

**DOI:** 10.4103/0972-9941.58499

**Published:** 2009

**Authors:** S Milanchi, I Makey, R McKenna, D R Margulies

**Affiliations:** Department of Surgery, Thoracic Surgery and Trauma, Surgical Director, Women's Guild Lung Institute, Program Director, General Thoracic Surgery Fellowship, Cedars-Sinai Medical Center, Los Angeles, California; 1 Medical Director, Thoracic Surgery and Trauma, Surgical Director, Women's Guild Lung Institute, Program Director, General Thoracic Surgery Fellowship, Cedars-Sinai Medical Center, Los Angeles, California

**Keywords:** Thoracotomy, trauma, video-assisted thoracoscopic surgery

## Abstract

**BACKGROUND:**

The role of video-assisted Thoracoscopic Surgery (VATS) is still being defined in the management of thoracic trauma. We report our trauma cases managed by VATS and review the role of VATS in the management of thoracic trauma.

**MATERIALS AND METHODS:**

All the trauma patients who underwent VATS from 2000 to 2007 at Cedars-Sinai Medical Center were retrospectively studied.

**RESULTS:**

Twenty-three trauma patients underwent 25 cases of VATS. The most common indication for VATS was retained haemothorax. Thoracotomy was avoided in 21 patients. VATS failed in two cases. On an average VATS was performed on trauma day seven (range 1-26) and the length of hospital stay was 20 days (range 3-58). There was no mortality. VATS was performed in an emergency (day 1-2), or in the early (day 2-7) or late (after day 7) phases of trauma.

**CONCLUSION:**

VATS can be performed safely for the management of thoracic traumas. VATS can be performed before or after thoracotomy and at any stage of trauma. The use of VATS in trauma has a trimodal distribution (emergent, early, late), each with different indications.

## INTRODUCTION

Thoracoscopy and video-assisted thoracoscopic surgery (VATS) is used increasingly by thoracic surgeons around the world and has become a standard procedure at many thoracic surgical centers. VATS is also used for the management of thoracic trauma. Thoracoscopy in trauma patients was first described in 1976[1] by Jackson and Ferreira, where thoracoscopy was used to diagnose diaphragmatic injuries in victims with penetrating injury to the left lower chest. In 1981, Jones *et al*.,[2] performed emergency thoracoscopy under local anesthesia on 36 trauma patients presenting with haemothoraces, which had high output from the chest tubes. Thoracotomy was avoided in 44% of these patients, based on the findings on thoracoscopy. VATS has been described post-trauma for the diagnosis and management of thoracic injuries (e.g., control of bleeding) and for the management of the complications of trauma (e.g., empyema or retained haemothorax). Compared to thoracotomy, VATS is reported to have fewer postoperative complications,[3] better postoperative pain control, fewer wound and pulmonary complications, shorter time to resumption of normal activity and shorter chest tube duration time.[4] The indications for VATS in trauma patients are shown in Table 1. In order to further evaluate the role of VATS in the management of trauma patients, we designed a retrospective study of the VATS cases performed on our trauma patients.

**Table 1:** Indications of video-assisted thoracoscopic surgery in trauma patients

Chylothorax
Diagnostic
Diaphragmatic injuries
Empyema
Foreign body in pleural space
Pericardial effusion
Persistent pleural effusion
Persistent bleeding from chest
Large haemothorax
Retained haemothorax

## MATERIALS AND METHODS

All the trauma patients who underwent VATS from 2000 to 2007 in a level-I urban trauma centre were retrospectively studied. No case was excluded. The patients' charts were reviewed for demographic data, mechanism of injury, indication for VATS, procedures performed, outcome, length of stay (LOS) in hospital and Injury Severity Score (ISS). The data was summarized and presented as a case series. The trauma patients who underwent thoracotomy during the same period were studied as well, and their data extracted from the trauma registry database.

## RESULTS

**Figure 1:**
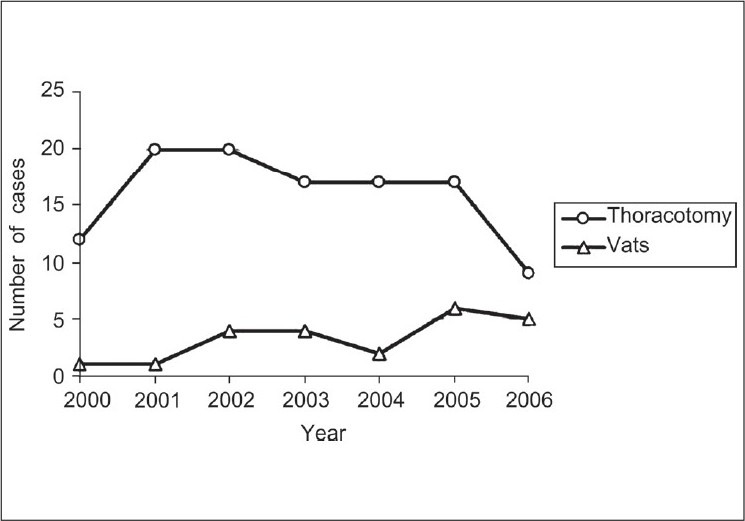
Number of trauma thoracotomies and video-assisted thoracoscopic surgery performed from 2000 to 2006. Note the decreasing number of thoracotomies and increase in the number of video-assisted thoracoscopic surgery cases

From 2000 to 2007, an average of 1,330 trauma patients was seen annually in our medical centre with a mean ISS of 9.5. Among those, 23 patients underwent a total of 25 VATS procedures. During the same time 112 thoracotomies were performed on trauma patients, including Emergency Room (ER) and Operating Room (OR) thoracotomies [Figure 1].

All the patients who underwent VATS were haemo dynamically stable at the time of the procedure and remained stable intraoperatively. The procedures were performed by cardio-thoracic surgeons in 24 cases and by a general surgeon in one case. Three patients (13%) were female and 20 (87%) were male. The mean age of the patients was 39 ± 15 years. The mechanism of injury was a gunshot wound (GSW) in nine patients, stab wound in five, motor vehicle collision (MVC) in eight and a fall in one. There was no mortality. The mean injury severity score was 21 ± 8.

### Indications and type of procedures

Fourteen patients underwent VATS for evacuation of retained haemothoraces. In this group VATS was performed on an average on trauma day six. Two of these patients had a second VATS during the same admission, two and nine days after the first VATS. Two patients underwent diagnostic VATS after stab wounds to the chest on trauma day one (i.e., day of trauma). Bleeding from an intercostal artery was controlled in one case and no intrathoracic injury was found in the other one. Two patients underwent VATS for pleural decortication, for empyema, on trauma days eight and 25. One was converted to thoracotomy due to extensive adhesions. One patient with avulsion of the right middle lobe bronchus after MVC underwent VATS for right middle lobectomy and bronchoplasty on trauma day two. One patient with pericardial effusion had VATS for pericardial window on trauma day 17, after a stab wound to the chest. VATS for ligation of the thoracic duct was performed for control of chylothorax in one patient on trauma day five, after MVC. VATS was performed for removal of knife from the chest in one case, on trauma day one. In another case sponges were removed by VATS 15 days after an ER thoracotomy for GSW to the chest.

### Thoracotomy and video-assisted thoracoscopic surgery

Five patients had thoracotomy performed before VATS, three of them being ER thoracotomies after GSW to the chest. Two of these three cases needed VATS for evacuation of retained haemothorax and the third one for retained sponge, as mentioned earlier. One patient had Operating Room thoracotomy for a large haemothorax and after five days underwent VATS for ligation of the thoracic duct for chylothorax. One case of VATS was performed for retained haemothorax five days after Operating Room median sternotomy in a victim of GSW, who sustained injuries to the heart, inferior vena cava (IVC) and lung.

One case of VATS performed for empyema 25 days after MVC, was converted to thoracotomy due to technical difficulties. Another patient needed thoracotomy three days after VATS for evacuation of retained haemothorax after MVC.

Sixteen patients were spared a thoracotomy and five spared a repeat thoracotomy because of VATS. A good example of the benefits of obviating thoracotomy was VATS, for evacuation of retained haemothorax in a 70-year-old lung transplant recipient, five days after an MVC. This patient could have had severe complications following a thoracotomy.

### Association with laparoscopy and laparotomy

Two victims of stab wounds had laparotomy and VATS on the day of trauma. One case who suffered from multiple thoracoabdominal stab wounds underwent laparotomy for repair of diaphragmatic and liver injuries, followed immediately by VATS. Another victim of stab wound to the chest underwent VATS, then diagnostic laparoscopy and subsequently laparotomy, for repair of gastric injury. Change of the patients' position from supine to lateral decubitus and vice versa was necessary in these cases, which were performed uneventfully.

### Timing of the procedure

The average LOS in the hospital was 20 days. On an average VATS was performed on trauma day seven. The average LOS in the hospital after VATS was 12 days. Four cases of VATS were performed emergently on trauma day one. Two of these were diagnostic after stab wound to the chest; one was performed for retained haemothorax after GSW, and one for removal of foreign body (knife) from the chest. The latest VATS was performed on trauma day 25 for empyema after MVC.

**Figure 2:**
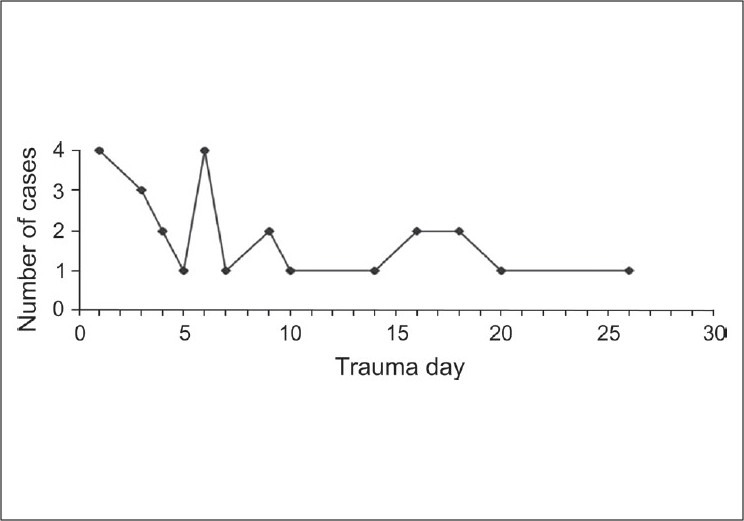
Distribution of video-assisted thoracoscopic surgery cases after trauma. Note the tri-modal distribution of the cases: Emergency, early and late

Based on the timing of performing VATS, we can divide our cases into three types: Emergency, early and late. Emergency cases were performed on arrival to the Emergency Room and on trauma day one or two, for diagnostic purposes, evacuation of retained haemothorax (after placement of chest tube), control of bleeding from intercostal arteries, or removal of foreign bodies from the chest. Early cases were performed from trauma day two to day seven for evacuation of retained haemothorax, control of bleeding (e.g., from the intercostal arteries), repair of bronchial injuries and ligation of injured thoracic duct. Late cases were performed after trauma day seven for retained haemothorax, empyema, pleural effusion, or pericardial effusion [Figure 2].

## DISCUSSION

The role of VATS in the management of thoracic injuries is expanding.[5] It is a valuable tool for the trauma surgeon and can obviate the need for thoracotomy and its associated complications. As described in the literature it can be used in an acute setting as a diagnostic or therapeutic tool as well as for managing the short-term or long-term complications of trauma. In stable trauma patients, VATS is safe and tolerated better by the patients than thoracotomy, with less postoperative complications.[4]

Haemodynamic stability was a prerequisite for doing VATS in our study, which is in agreement with the current literature. Our study shows that VATS can be performed safely in patients who had thoracotomy before VATS; this is not previously described in the literature. Our study shows that VATS can be performed regardless of the mechanism of injury, whether blunt trauma, GSW, stab wound, MVC, fall, or assault. Our rate of conversion to thoracotomy was 4% (one patient), as well as rate of failure of VATS. VATS can be performed for removal of foreign body from the chest (e.g., knife after stab wound) or removal of a retained foreign body (e.g., retained gauze). VATS can be performed safely in association with laparotomy or laparoscopy, immediately, before, or after them. Most of the current literature about VATS in trauma patients is from centers outside the U.S.[6–8] and although this technique is known to the trauma surgeons for three decades it is still not widely used for the management of thoracic trauma in the U.S. There are barriers to the widespread use of VATS in trauma; among them are lack of training of the trauma surgeons, limited access of trauma surgeons to thoracic surgical equipment due to hospital policies, large volume of trauma patients in trauma centers and financial problems. By overcoming these barriers, trauma surgeons can add VATS to their armamentarium.

## CONCLUSIONS

We have found that VATS is a safe and effective procedure in haemodynamically stable patients in subacute settings, who have blunt or penetrating traumas to the chest. VATS can be performed in patients who have thoracotomy and it can be performed in association with laparotomy or laparoscopy. With evolving experience, further studies will be needed to establish its safety and usefulness in acute settings.
